# Reconstruction of Bone Defect Combined with Massive Loss of Periosteum Using Injectable Human Mesenchymal Stem Cells in Biocompatible Ceramic Scaffolds in a Porcine Animal Model

**DOI:** 10.1155/2019/6832952

**Published:** 2019-11-23

**Authors:** Chun-Cheng Lin, Shih-Chieh Lin, Chao-Ching Chiang, Ming-Chau Chang, Oscar Kuang-Sheng Lee

**Affiliations:** ^1^Institute of Clinical Medicine, National Yang-Ming University, Taipei 11221, Taiwan; ^2^Division of Orthopaedic Trauma, Department of Orthopaedics and Traumatology, Taipei Veterans General Hospital, Taipei 11217, Taiwan; ^3^Department of Pathology and Laboratory Medicine, Taipei Veterans General Hospital, Taipei 11217, Taiwan; ^4^Department of Surgery, National Yang-Ming University, Taipei 11221, Taiwan; ^5^Department of Orthopaedics and Traumatology, Taipei Veterans General Hospital, Taipei 11217, Taiwan; ^6^Department of Medical Research, Taipei Veterans General Hospital, Taipei 11217, Taiwan

## Abstract

Clinically, in patients who sustain severe open fractures, there is not only a segmental bone defect needed to be reconstructed but also insufficient healing capacity due to concomitant damages to the periosteum and surrounding soft tissues. For studying the reconstruction of bone defects associated with massive loss of periosteum and surrounding soft tissues, there are no well-established preclinical models in large animals in the literature. The purpose of the study was to generate a large animal model of bone defect with massive periosteum loss and to adopt a tissue engineering approach to achieve rapid bony union with stem cells and biomaterials. In this study, a bone defect with massive periosteum stripping was generated in pigs, which was followed by emptying nearby canal marrow including fat and cancellous bone. The stripped periosteum was a mimic to the situation in the Gustilo type 3 open fractures. Bone defects were then reconstructed by impacting the biocompatible ceramic scaffold, morselized tricalcium phosphate (TCP) loaded with human adipose tissue-derived mesenchymal stem cells (hMSCs). Radiological and pathological assessments indicated that TCP and hMSCs synergistically promoted bone healing with increased lamination and ingrowth of vessels. Both bridging periosteum formation and gap filling were induced rapidly. In conclusion, a porcine model of segmental bone loss with damage of surrounding periosteum was created. Reconstruction of such defects with hMSCs and TCP achieved rapid union of bone defects associated with massive periosteal stripping.

## 1. Introduction

### 1.1. Background

Clinically, chronic defect or delayed union of long bones after fractures leads to subsequent complications such as muscle atrophy, joint stiffness, compromised limb function, and poor quality of life. In particular, in the patients who sustain Gustilo type 3 open fractures, there is not only a segmental bone defect needed to be reconstructed but also insufficient healing capacity due to vascular insufficiency to overcome. Regarding bone defects with massive loss of periosteum, there are no well-established preclinical models in large animals in the literature.

Angiogenesis is critical for bone fracture healing. During the formation of the bone callus, the vascular system plays an important role in promoting bone regeneration by supplying oxygen, nutrients, and ions necessary for mineralization through the formation of new vessels. The importance of blood vessels in delivering osteoprogenitors to the fracture site has also been recognized [1]. Besides angiogenesis, perivascular cells in the periosteum are suggested to be also a source of osteoprogenitor cells during periosteal osteogenesis [2].

Mesenchymal stem cells (MSCs) are multipotent cells of mesodermal origin capable of differentiating into osteoblasts, chondrocytes, adipocytes, tenocytes, and myoblasts [3]. New bone formation is dependent on the presence of MSCs at the location of bone damage. MSCs are the source of osteogenic lines of cells capable of forming bone matter. For nonunion cases, loading MSCs on an injectable carrier have been tested for efficacy as an alternative for open surgical procedures [4, 5]. As to critical bone defect, MSCs could facilitate osteogenesis if loaded in scaffolds of predefined dimensions and shape to fit in the defect [6].

Transplantation of human MSCs (hMSCs) in a nonautogenous setting for bone regeneration in a rabbit critical-sized defect (15 mm) has been reported [7]. In human studies, the combination of autologous hMSCs and hydroxyapatite granules was reported as a safe method for treating nonunion. The patients treated with hMSCs had faster initial radiographic and functional improvements [8]. Although hMSCs could facilitate osteogenesis when loaded in scaffolds of predefined dimensions and shape to fit in the defect, the efficacy of such approach in a poorly vascularized environment remains unknown.

hMSCs possess immunosuppressive potentials, and the concept of using universal donor cells for regenerative medicine is more clinically feasible. However, clinical translation is dependent on preclinical studies in animal models. The choice of animal model for studying functions of hMSCs hinges primarily on the methods to be used and the desired proximity to clinical translation [9]. Although there were some mild immune responses reported in the literature [10], the therapeutic effect of allogeneic hMSCs in bone repair was not significantly affected.

### 1.2. Objective and Hypothesis

For the treatment of segmental bone defects with massive periosteum loss, the critical issue is the formation of both vessels and new bone bridging across the gap between the fractured bone ends. Implant fixation with only bone substitutes is not adequate to achieve rapid union in patients with insufficient healing capacity due to microcirculation compromise. Bone bridge formation can be achieved by impacting morselized tricalcium phosphate, a biocompatible ceramic scaffold commonly used in orthopedic surgeries. In addition, it may be loaded with hMSCs in this challenging situation. It is also important to determine whether angiogenesis would be induced by this approach, as the induction of angiogenesis by hMSCs is a critical step to achieve new bone formation.

In order to evaluate the effects of transplanted MSCs in the bone defect with poor microcirculation, the periosteum surrounding the defect will be stripped off and the nearby canal marrow is also emptied in the animal model. The hypothesis of the study is that hMSCs loaded onto biocompatible ceramic scaffolds may induce osteogenesis and angiogenesis during reconstruction for segmental bone defects combined with massive loss of periosteum.

## 2. Materials and Methods

### 2.1. hMSCs

Adipose tissue-derived hMSCs were purchased from Steminent Biotherapeutics Inc. (Taipei, Taiwan). hMSCs were maintained in an expansion medium (MesenPRO RS™, Invitrogen, Grand Island, NY, USA) supplemented with 100 units/mL of penicillin, 1000 units/mL of streptomycin, and 2 mmol/L of L-glutamine (Sigma-Aldrich, St. Louis, MO, USA) and incubated at 37°C, 5% CO_2_, and 95% relative humidity. For inducing osteogenic differentiation, hMSCs were cultured with an osteogenic induction medium (OIM) consisting of Iscove's Modified Dulbecco's medium (Gibco, Grand Island, NY, USA) supplemented with 10 mM *β*-glycerol phosphate (Sigma-Aldrich, St. Louis, MO, USA), 0.1 *μ*M dexamethasone (Sigma-Aldrich, St. Louis, MO, USA), and 0.2 mM ascorbic acid (Sigma-Aldrich, St. Louis, MO, USA), and the medium was changed every three days.

### 2.2. The Bone Defect and Massive Loss of Periosteum

**Figure 1:**
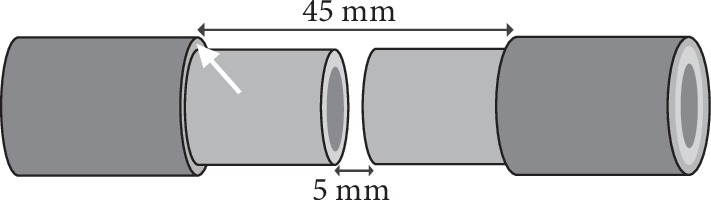
The design of each defect with massive periosteum loss. The periosteum (white arrow) was stripped totally 45 mm with one 5 mm bone defect in the center.

The porcine tibia's shape in the study was like a cylinder with about 12 mm in diameter. Each gap made on the pig's tibia shaft was 5 mm in length. Thus, the calculated volume in each bone defect was about 0.565 cc. Around the gap, the length of the stripped periosteum was 20 mm on each end of the fracture (Figure 1).

### 2.3. Tricalcium Phosphate

Commercially available morselized tricalcium phosphate (TCP) was used in this study (chronOS, Synthes, Inc., USA). The TCP used in this study contained irregular particles with 60% interconnected porosity in structure, and the average size of each particle was 2.8 to 5.6 mm in length and about 0.1 cc in volume.

### 2.4. The Pigs

Yorkshire pigs were used in this study. The age was about 2-3 months, with 14-15 kg in body weight. They were kept in individual cages, fed a standard diet, and allowed free activity during the study. Animal procedures were approved by the university committee on the use and care of animals at the National Yang-Ming University. The reason of using young Yorkshire pigs is the similarity in geometry of long bones to that of humans.

### 2.5. Design of Experiments

#### 2.5.1. Establishment of Surgical Fixation in the Animal Model

All animal protocols were subject to institutional approval and were carried out in compliance with licensing guidelines. The pigs were anesthetized with isoflurane inhalational anaesthesia and monitored by a technician during surgery. The biocompatible ceramic scaffold used was morselized TCP. Fixation of tibial bone defects was achieved by metallic plates and screws (Synthes, Inc.).

#### 2.5.2. Establishing the Segmental Defect in Long Bone with Poor Circulation

The segmental bone and surrounding periosteum were removed, and the sizes and location were as mentioned as above. The length of periosteum loss was 9 times longer than that of bone defect (Figure 1). Once the bone defect was obtained, the nearby canal marrow, including fat and cancellous bone, was also emptied. The segmental bone defects were supposed to have poor vascular supplement as both periosteal and intramedullary microcirculations were compromised.

#### 2.5.3. Establishing the Control Defect and Treatment of Defects

The control defect (C) was generated by removal of bony gap, surrounding periosteum and nearby marrow. TCP was impacted in the defect. The treatment defects were divided into 2 groups, named A and B. Then, each group was divided into 2 subgroups, A1/A2 or B1/B2. In treatment groups, only defect A1 had preserved periosteum.

**Figure 2:**
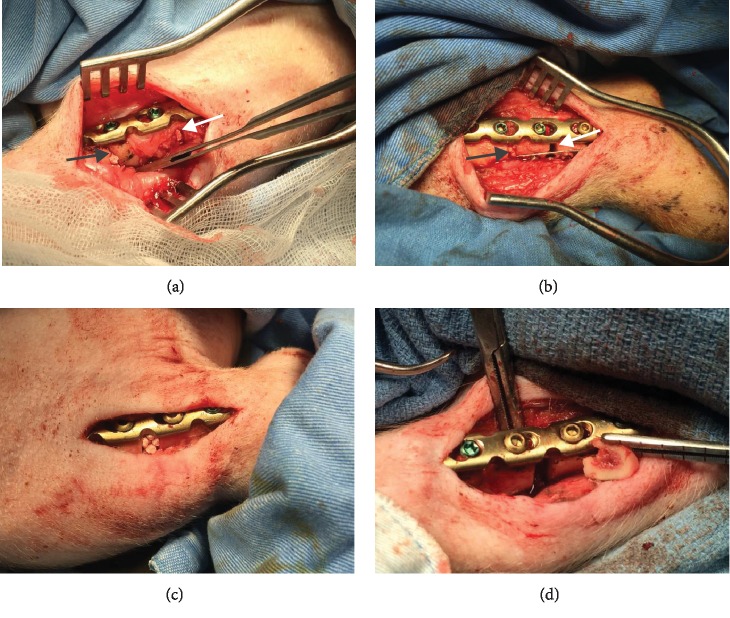
Models of defects C/A1, C/A2, B1, and B2 and photography. (a) The *Tibia 1* has control defect C (black arrow) with stripped periosteum whereas the treatment defect A1 (white arrow) with preserved periosteum. Both defects C and A1 have tricalcium-phosphate impacted. (b) The *Tibia 2* has control defect C (black arrow) impacted with tricalcium phosphate, and treatment defect A2 (white arrow) without any implantation. In this group, the length of trimmed periosteum was totally 70 mm for two lesions (overlapping 20 mm). (c) The *Tibia 3* has periosteum-stripped defect B1 with tricalcium phosphate impacted followed by injection of human mesenchymal stem cells. (d) The *Tibia 4* has periosteum-stripped defect B2 without tricalcium phosphate. Human mesenchymal stem cells are then loaded into the empty space of defect B2.

The authors designed that defects of C/A1 (named as Tibia 1) (Figure 2(a)) or C/A2 (named as Tibia 2) (Figure 2(b)) were in the same tibia shaft of individual pig. Thus, in the Tibia 2, the length of trimmed periosteum was totally 70 mm for two lesions (overlapping 20 mm) in one tibia. But defects B1 and B2 were separately in different tibia shafts (named as Tibia 3 and 4) (Figures 2(c) and 2(d)). The A1 defect had TCP impacted in the gap, whereas A2 was totally empty. The B1 defect had hMSC-loaded TCP in the gap, whereas B2 had only hMSCs (Table 1).

#### 2.5.4. Assessment of Osteogenesis and Angiogenesis

The roentgenographic and histopathological assessments were performed 4 and 8 weeks after the operation. The X-rays were obtained by the portable C-arm (Siemens, Inc., Germany). Tissue biopsy was performed 12 weeks later postoperatively. The bone tissues were obtained from the middle of the gap. The radiographical findings were graded by the Lane-Sandhu scoring system (Table 2) [11].

The hematoxylin-eosin stain was used in histopathological assessment. Under microscopy, the gross numbers of osteocytes, phenomenon of bone lamination in osteocytic area, and vessels in the marrow tissue area were observed.

## 3. Results

**Figure 3:**
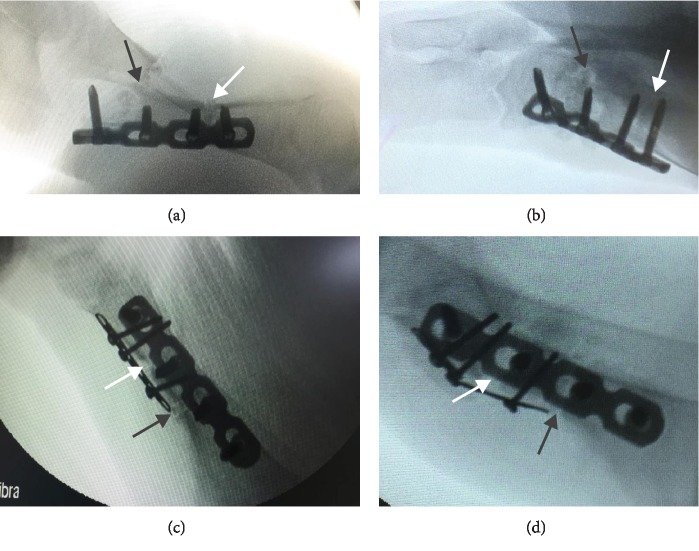
Models of defects C, A1, and A2 and radiography. Time-dependent radiological changes in the defect, 4 and 8 weeks after reconstruction. (a) 4 weeks after reconstruction, the *Tibia 1* showing failed defect C (black arrow) and callus formation between the cortex gap of defect A1 (white arrow). (b) 8 weeks after reconstruction, much more callus connecting two ends of defect A1. (c) 4 weeks after reconstruction, the *Tibia 2* showing more callus formation in control defect C (black arrow) than in treatment defect A2 (white arrow). (d) 8 weeks after reconstruction, broken plate happens, whereas more callus in defect C.

**Figure 4:**
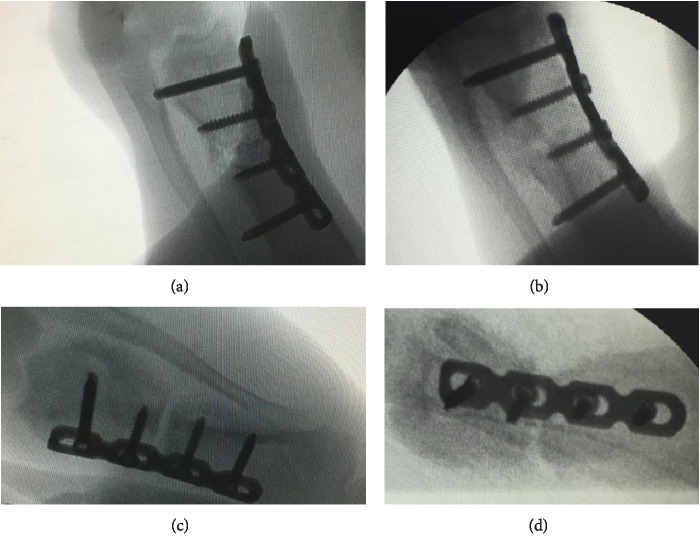
Models of defects B1 and B2 and radiography. Time-dependent radiological changes in the defect, 4 and 8 weeks after reconstruction. (a, b) 4 and 8 weeks after reconstruction, the *Tibia 3* showing bone bridge connecting the defect B1. (c, d) 4 and 8 weeks after reconstruction, the *Tibia 4* showing overgrowth of callus upon bilateral ends aside the defect B2, without crossing the gap.

**Figure 5:**
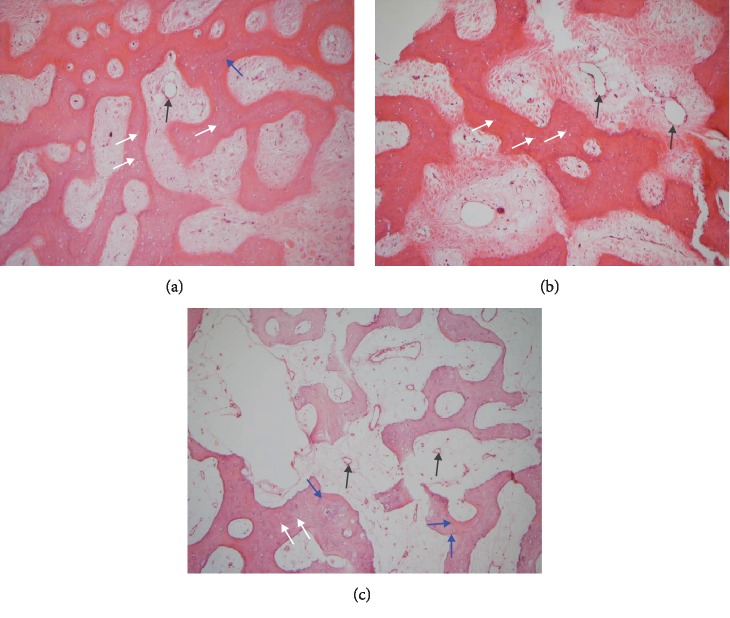
Models of defects C, A1, and A2 and histology. The pathological section stained with hematoxylin-eosin stain 12 weeks after reconstruction. White arrows indicate osteocytes, blue arrows indicate lamination, and black arrows indicate vessels in the marrow region. (a) The control defect C of the *Tibia 2*, showing many osteocytes but seldom vessels and lamination. (b) The treatment defect A2 of the *Tibia 2*, no apparent lamination formation. (c) The treatment defect A1 of the *Tibia 1*, less osteocytes than defect C, but much more lamination.

**Figure 6:**
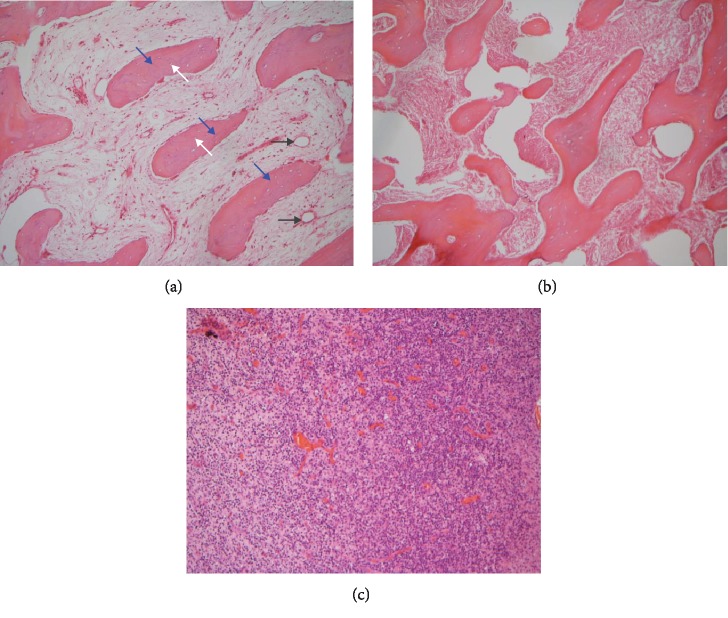
Models of defects B1 and B2 and histology. The pathological section stained with hematoxylin-eosin stain 12 weeks after reconstruction. White arrows indicate osteocytes, blue arrows indicate lamination, and black arrows indicate vessels in the marrow region. (a) The treatment defect B1 of the *Tibia 3*, showing apparent lamination like as defect A1. (b) The section obtained from one end of the treatment defect B2, showing normal bone formation. (c) The section obtained in the middle of the treatment defect B2 of the *Tibia 4*, showing granulation tissue.

All of the results were sorted in descending power, respectively, in assessments of radiographic images and pathological sections, except the control defect C of Tibia 1 and the treatment defect B2 of Tibia 4. The control defect C of Tibia 1 was failed due to loosening of plate (Figures 3(a) and 3(b)). In the Tibia 2, double plates (Figures 3(c) and 3(d)) were performed, and bone alignment was kept during follow-up time. A single locking plate was applied, respectively, to Tibia 3 (Figures 4(a) and 4(b)) and Tibia 4 (Figures 4(c) and 4(d)). As for treatment defect B2, overgrowth of bone callus was found by C-arm fluoroscopy, but it occurred upon bilateral ends aside the defect, without crossing the gap. In the histopathological assessment, much more osteocytes were found in the control defect C and A2 (Figures 5(a) and 5(b)) than defects A1 (Figure 5(c)) and B1 (Figure 6(a)). However, more vessels and lamination were found in defects A1 and B1 than C and A2. The biopsy of callus from one side of defect B2 showed normal bone formation (Figure 6(b)), but the section obtained inside the middle of the gap only revealed granulation tissues under a light microscope (Figure 6(c)). The appearance of Tibia 4 was much more swelling than that of normal legs. The Tibia 4 was also the only limping leg in the 12^th^ week of follow-up. The defects, including control defect C of Tibia 2, treatment defects A1, A2, and B1, were sorted (Table 3) according to the results from the Lane-Sandhu score, numbers of osteocytes, maturation of bone lamination, and numbers of vessels in the marrow area. The treatment defects A1 and B1 had the closest results. There was no complication of infection nor systemic allergy during the whole course of follow-up.

## 4. Discussion

### 4.1. Bone Defect with Compromised Circulation

Reconstructing bone defects with compromised circulation is one of the most challenging conditions for orthopedic surgeons. In this situation, the loss of bone and soft tissues perturbs normal fracture healing processes, which requires intervention to prevent the occurrence of nonunion. Massive loss of periosteum and surrounding soft tissues compromises the vascularization and healing capacity.

In the animal model reported in this study, a massive periosteum defect was obtained, followed by emptying nearby canal marrow including fat and cancellous bone. The situation of stripped periosteum was like in Gustilo type 3 open fractures. As for the removal of nearby canal marrow, it is an important procedure to minimize the compensation from the intramedullary circulation.

### 4.2. The Osteogenic Lines of Cells in Application to Bone Healing

Regenerative medicine using stem cell therapy has sparked much interest in this 21^st^ century not only because of the controversies that surround the ethics involving pluripotent stem cells but also because of their potential for use in the clinic. The complex interactions between the soft tissues surrounding the bone defect, periosteum, and the bone marrow are based on a yet only partially understood interplay between different cell types [12]. Due to limitations associated with autografts and bank-stored allografts, investigators have alternative solutions such as biodegradable ceramics. Scaffolds are optimized for a permanent support for new cells and tissue, or a temporary absorbable cell delivery system. Attention of contemporary research is focused on the development of three-dimensional (3D) scaffolds planted with stem cells that might be used for the reconstruction of extensive bone defects. A 3D matrix with an open, porous structure (100-1000 *μ*m) allows space both for cells to attach for nutrients and waste to permeate, and also room for extracellular matrix (ECM) accumulation [13]. Synthetic biodegradable ceramics based on hydroxyapatite (HA) and TCP show good incorporation into bone tissue due to their biocompatibility and porous structure. However, the lack of resorption of HA may compromise the biomechanics of the newly formed bone over time [14]. Polyesters of naturally occurring alpha-hydroxy acids, such as poly glycol acid (PGA) and polylactic-co-glycolic acid (PLGA), are also widely used in tissue engineering. But polymers have lower ability of osteointegration compared to biodegradable ceramics, and their degradation is connected with stronger tissue reaction [15]. Another concept is the anatomically shaped scaffolds which are generated from fully decellularized trabecular bone by using digitized clinical images and seeded with MSCs [12]. Tricalcium phosphate was chosen in the current porcine model for its good resorption and clinical availability.

### 4.3. Compared with Contemporary Clinical Strategies for Segmental Bone Defect Healing

The results of the study could prevent some disadvantages from recently clinical reconstruction methods for segmental bone defect healing such as autologous bone graft, allogenic strut bone graft, or distraction histeogenesis.

Autologous bone grafts from the iliac crest are considered to be the gold standard [16], but the shape and size limit ideal reconstruction for a large segmental defect. The other disadvantage is the subsequent donor site pain and morbidity [17]. On the contrary, allogenic strut bone graft supports biologic reconstruction of segmental bone defects. However, bone callus just occurs surrounding the junction line between the host bone and the allograft. Besides, instability and subsequent implant failure due to prolonged process of fracture union are still commonly seen. Additional augmentation with autologous sponge bone graft or bone morphogenetic proteins (BMPs) may enhance the allogenic bone healing, but clinical results are still not ideal and the optimal timing and dosage of administration of BMPs remain elusive [18–21]. As for distraction histeogenesis, only Ilizarov procedure is capable of stimulating osteogenesis and angiogenesis for bone reconstruction simultaneously [22, 23]. The living bone regenerated in distraction osteogenesis will eventually provide sufficient biomechanical strength, length, and durability [24]. Nevertheless, the difficulties in techniques and prolonged external fixation may lead to complications such as skin invaginations, pes equinus, pin-tract infection, or premature consolidation of the regenerate [25].

### 4.4. Challenges in the Current Study

In clinical practice, controlling nonweight bearing or partial-weight bearing for the treated limb was possible according to the patient's compliance. However, in the porcine study, free movement of the experimental animals after surgeries resulted in implant failure in some of the animals. Another issue was the choice of implants. Plating fixation was supposed to be better when concerning that the space of bone defect could be occupied by intramedullary nail and not suitable for grafting. The reason for designing two defects (without hMSCs) in the same tibia was to minimize surgery frequency and numbers of experimental pigs. But implant failure was noted in the Tibia 1 where the screw's pulling out occurred aside the defect C. Thus, the control defect C in Tibia 1 was not available for subsequence analysis. Given the failed plate in the Tibia 1, the authors used double plates for the Tibia 2, one locking plate like as that in Tibia 1 and further augmentation with another miniplate. Although the miniplate was then broken, the alignment of Tibia 2 was still kept by the locking plate during the follow-up time. Rather than Tibia 1 and 2, the treatment defects B1 and B2 were, respectively, made in different tibia. Grafting hMSCs into a single defect on one tibia was to avoid the impaction brought by unpredictable migration of stem cells.

In the groups with impacted TCP, it was difficult to differentiate callus formation from tricalcium phosphate by C-arm fluoroscopy. The TCP resorption was unpredictable. It was supposed that the reliable indicator to evaluate bone healing was the callus around the defect, especially with the sign of crossing the gap. The treatment defect B1 had the most similar pattern of bone bridge when compared with treatment defect A1 which had preserved periosteum and impacted TCP in the defect. As for treatment defect B2 which was implanted with MSCs, apparent bone callus covered bilateral ends of the defect, but there was no sign of bone bridge crossing the gap. In the view of reconstruction, the procedure in the treatment defect B2 was a failure.

Using a light microscope and calculating numbers of osteocytes were not good ways to evaluate healing capacity in each treated defect because the remodeling architecture of new bone was irregular and the space in the defect was supposed to be partially occupied by impacted TCP. Therefore, the maturation of lamination was much more specific for bone healing than the numbers of osteocytes in the field of transverse view.

### 4.5. Novel Achievement of the Study

The novel achievement of the study was to provide evidence of angiogenesis and osteogenesis promoted by injectable human MSCs as a form of allogeneic graft. In the literature, it has been reported that angiogenesis occurred prior to osteogenesis for the osteogenic differentiation of MSCs. However, engineered vascularization in the TCP was time-consuming and may not be practical for clinical reconstruction [26]. Based on the findings of this study, angiogenesis has been achieved in massive periosteum defect which was 9 times more than the length of the bone defect.

### 4.6. Limitations of the Study

Although the stem cells trapped by TCP could be inferable upon the bone healing results found in treatment defects B1 (hMSCs with TCP) and B2 (hMSCs without TCP), there was no definitive evidence. Further analysis by microtomographs and long-term staining of stem cells should be enrolled, respectively, for day-by-day healing process and cell recognition.

## 5. Conclusions

In this porcine model, simultaneous new bones for bridging periosteum and filling bone gap were achieved. TCP and hMSCs cooperatively promoted bone healing with increased lamination and vessels. Further clinical translation may be warranted.

## Figures and Tables

**Table 1:** The characteristics of defects.

Group (*N* = 1 for each group)	Periosteum	Defect	Implantation
Tibia 1	Trimmed	C	TCP
	Preserved	A1	TCP
Tibia 2	Trimmed	C	TCP
	Trimmed	A2	Empty
Tibia 3	Trimmed	B1	TCP+hMSCs
Tibia 4	Trimmed	B2	hMSCs

**Table 2:** The Lane-Sandhu score.

0	No callus
1	Minimal callus formation
2	Callus evident and beginning osseous formation
3	Callus evident and fracture line almost obliterated
4	Complete union with complete remodeling

**Table 3:** Results of assessments sorted in descending power.

Time of follow-up (weeks)	Assessments	
	*C-arm fluoroscopy* (*Lane-Sandhu score*)	
4	B1=A1>C>A2 (2=2>1>0)	
8	B1=A1>C>A2 (3=3>2>1)	

	*Pathological microscope*	
12	*Osteocyte numbers*	A2>C>B1≈A1
	*Lamination*	B1≈A1>C>A2
	*Marrow vessels*	B1≈A1>A2>C

The defects (including control defect C of Tibia 2, treatment defects A1, A2, and B1) were sorted according in descending power to the results from the Lane-Sandhu score, numbers of osteocytes, maturation of bone lamination, and numbers of vessels in the marrow area. The treatment defects A1 and B1 had the closest results.

## Data Availability

The data that support the findings of this study are available from the corresponding author upon reasonable request.
